# GAS6/TAM signaling pathway controls MICA expression in multiple myeloma cells

**DOI:** 10.3389/fimmu.2022.942640

**Published:** 2022-07-28

**Authors:** Andrea Kosta, Abdelilah Mekhloufi, Lorenzo Lucantonio, Alessandra Zingoni, Alessandra Soriani, Marco Cippitelli, Angela Gismondi, Francesca Fazio, Maria Teresa Petrucci, Angela Santoni, Helena Stabile, Cinzia Fionda

**Affiliations:** ^1^ Department of Molecular Medicine, Sapienza University of Rome, Rome, Italy; ^2^ Department of Biomedical Engineering, Emory University, Atlanta, GA, United States; ^3^ Division of Hematology, Department of Translational Medicine and Precision, Sapienza University of Rome, Rome, Italy; ^4^ Istituto di Ricovero e Cura a Carattere Scientifico (IRCCS) Neuromed, Pozzilli, Italy; ^5^ Istituto Pasteur-Fondazione Cenci Bolognetti, Sapienza University of Rome, Rome, Italy

**Keywords:** GAS6, AXL, MERTK, MICA, NKG2D ligand, multiple myeloma, natural killer cells, bone marrow stromal cells

## Abstract

NKG2D ligands play a relevant role in Natural Killer (NK) cell -mediated immune surveillance of multiple myeloma (MM). Different levels of regulation control the expression of these molecules at cell surface. A number of oncogenic proteins and miRNAs act as negative regulators of NKG2D ligand transcription and translation, but the molecular mechanisms sustaining their basal expression in MM cells remain poorly understood. Here, we evaluated the role of the growth arrest specific 6 (GAS6)/TAM signaling pathway in the regulation of NKG2D ligand expression and MM recognition by NK cells. Our data showed that GAS6 as well as MERTK and AXL depletion in MM cells results in MICA downregulation and inhibition of NKG2D-mediated NK cell degranulation. Noteworthy, GAS6 derived from bone marrow stromal cells (BMSCs) also increases MICA expression at both protein and mRNA level in human MM cell lines and in primary malignant plasma cells. NF-kB activation is required for these regulatory mechanisms since deletion of a site responsive for this transcription factor compromises the induction of *mica* promoter by BMSCs. Accordingly, knockdown of GAS6 reduces the capability of BMSCs to activate NF-kB pathway as well as to enhance MICA expression in MM cells. Taken together, these results shed light on molecular mechanism underlying NKG2D ligand regulation and identify GAS6 protein as a novel autocrine and paracrine regulator of basal expression of MICA in human MM cells.

## Introduction

Natural Killer (NK) cells are innate lymphocytes playing a prominent role in antitumor-immune response (1, 2). A complex repertoire of activating and inhibitory receptors controls their ability to recognize and kill target cells (3). Because high tumor-associated expression of ligands MICA, MICB and ULBPs, the stimulatory receptor NKG2D strongly contributes to NK cell response against solid and hematologic cancers (4, 5), including multiple myeloma (MM) (6), a neoplasia caused by accumulation of malignant plasma cells (PCs) in the bone marrow (BM). Advanced-stage MM is associated with defective recognition of cancerous PCs and suppression of NK cell function (7). Indeed, promising strategies to treat this incurable cancer include NK cell therapeutic approaches (8, 9). To this aim, several studies have focused on the comprehension of mechanisms underlying the expression of NKG2D ligands on MM cells. Of note, a number of oncoproteins, such as Hsp90, STAT3, IRF4, C-MYC and IKAROS/AIOLOS, emerged as negative regulators of *MICA* gene transcription (10–13). However, pathways driving basal expression of these ligands on MM cells remain largely unexplored. It is also completely unknown whether BM microenvironment plays a role in these mechanisms. The survival, proliferation and drug resistance of malignant PCs depend on autocrine and paracrine loops involving many cellular and non-cellular components of BM (14, 15). In particular, adhesive interaction as well as soluble factors released by BM stromal cells (BMSCs) strongly promote MM pathogenesis and progression (16). Our recent findings identified BMSCs as regulators of NK cell anti-MM response. We reported a role for IL-8-bearing microvesicles released by BMSCs in the regulation of the expression of Poliovirus receptor (PVR/CD155) (17), a ligand of the receptors DNAM-1, TIGIT and TACTILE/CD96 expressed by NK cells (18, 19).

Accumulating evidence demonstrated the contribution of GAS6/TAM pathway as an oncogenic signaling in MM pathogenesis (20–23). The TAM subfamily of tyrosine kinase receptors (RTK) includes TYRO3, AXL and MERTK (TAM) which are able to bind two vitamin-K dependent proteins, Growth arrest-specific gene 6 (GAS6) and Protein S (PROS1). Ligand binding induces dimerization and autophosphorylation of TAM receptors, followed by activation of many downstream pathways like MEK/ERK, PI3K/AKT and JAK/STAT signaling (24, 25).

MM cells express TAM receptors, mainly MERTK, and produce high amount of GAS6, which is also largely secreted by BMSCs (20, 22). In MM cells, TAM receptor activation triggers MAPK/ERK, PI3K/Akt and NF-kB signaling and promotes the survival and proliferation. Blockade of MERTK on MM cells or pharmacologic targeting of GAS6 reduces myeloma burden and increases survival of mice bearing an orthotopic myeloma model (22). Such effects support a role for GAS6/TAM receptors as novel therapeutic targets for this malignancy.

Importantly, TAM pathway can exert a direct regulatory activity on diverse immune cells, including NK cells. It is required for NK cell development and functional maturation in human and mice (26, 27). NK cells isolated from TAM-deficient mice have poor cytotoxic activity because of impaired expression of inhibitory and activating receptors. On the other hand, TAM-mediated signaling was shown to attenuate NK cell response in mouse metastasis models in a Cbl-b-dependent manner (28, 29). More recently, it was reported that activated human NK cells rapidly acquired TYRO3 from tumor cells *via* trogocytosis and become more cytotoxic (30).

However, the role of GAS6 and its receptors TYRO3, AXL and MERTK in the regulation of activating ligands and immunosurveillance of MM is unknown.

In this study, we investigated the impact of GAS6/TAM pathway on NKG2D-dependent recognition of MM cells. We demonstrated that defective TAM signaling causes a selective reduction of surface MICA expression on MM cells, compromising their ability to induce NKG2D-dependent NK cell degranulation. We also found that BMSC-derived GAS6 can promote the expression of this NKG2D ligand on MM cell lines and primary PCs *via* activation of NF-kB pathway.

Our findings provide evidence of a role for GAS6 protein as a novel autocrine and paracrine regulator of basal expression of MICA in human MM cells.

## Materials and methods

### Cell lines and clinical samples

Human MM cell lines SKO-007(J3) and ARP-1 were kindly provided by Prof. P. Trivedi (Sapienza, University of Rome, Italy). The human MM cell line U266 was kindly provided by Prof. Nicola Giuliani (University of Parma, Italy). These cell lines were maintained at 37°C and 5% CO_2_ in RPMI 1640 (Life Technologies, Gaithersburg, MD) supplemented with 10% FCS, and were authenticated by IRCCS Azienda Ospedaliera Universitaria San Martino-IST, S.S. Banca Biologica e Cell factory (Genova, IT). The human 293T embryonic kidney cells were purchased from ATCC (Manassas, VA) and were maintained in Dulbecco’s modified Eagle’s supplemented with 10% FCS. All cell lines were mycoplasma-free (EZ-PCR Mycoplasma Test Kit, Biological Industries).

Bone marrow (BM) samples from MM patients were managed at the Division of Haematology, Department of Translational Medicine and Precision, Sapienza University of Rome. Informed consent in accordance with the Declaration of Helsinki was obtained from all patients, and approval was obtained from the Ethics Committee of the Sapienza University of Rome (RIF.CE: 5191). BM aspirates were processed as described in (31). MM cells were selected using anti-CD138 magnetic beads (Miltenyi Biotec, Auburn, CA, USA). More than 95% of the purified cells expressed CD138 and CD38. Bone Marrow Stromal Cells (BMSCs) were obtained from CD138^-^ fraction, phenotypically characterized and assessed for their capacity of osteogenic and adipogenic differentiation as previously described (17).

### Reagents and antibodies

Monoclonal antibodies (mAbs) anti-CD138/FITC, anti-CD38/APC, anti-CD107a/APC, anti-CD45/Pecy7, anti-CD3/APC-H7, CD14/APC-H7, CD19/APC-H7 anti-CD56/BV421, anti-MERTK/BB700 (125518) and anti-Axl/BV711 (108724) were purchased from BD Biosciences (San Jose, California, USA). Unconjugated mAbs anti-MICA (MAB159227), anti-MICB (MAB236511), anti-ULBP1 (MAB170818), anti-ULBP2/5/6 (MAB165903), anti-ULBP3 (MAB166510), anti-NKG2D (MAB149810), anti-B7/H6 (MAB71444), anti-ICAM-1 (BBA3), anti-MICA/AF488 (MAB159227) and anti-Tyro3/AF488 (FAB859G) conjugated mAbs were all purchased from R&D System (Minneapolis, USA). Anti-PVR (SKII.4) was kindly provided by Prof. M. Colonna (Washington University, St Louis, MO). Anti-MHC class I (W6/32) mAb was purchased from ATCC (Manassas, Virginia, USA). Allophycocyanin conjugated with goat anti mouse (GAM-APC) (Poly4053) mAb was purchased from Jackson Immuno-Research Laboratories (Cambridgeshire, UK). Anti-GAS6 (D3A3G) mAb, anti-phosphorylated p65, anti-phosphorylated AXL and anti-AXL were purchased from Cell Signaling Technology (Danvers, MA, USA). Anti-p65 was purchased from Santa Cruz Biotechnologies (Dallas, TX, USA). Anti-β-Actin (AC-15) mAb was purchased from Sigma-Aldrich (St. Louis, MO, USA). Donkey anti-rabbit (NA934V) or sheep anti-mouse (NA931V) secondary Hrp-linked mAbs were purchased from GE Healthcare (Wisconsin, MA, USA). The inhibitor UNC2250 was purchased from Selleck (Houston, TX, USA).

### Flow cytometry and degranulation assay

MM cell lines (1,5 x 10^5^ cells/mL) were cultured for 72h in complete medium or in BMSC-conditioned medium (BMSCs-CM). Cells were stained with anti-MICA mAb followed by secondary goat anti-mouse APC Ab. In all experiments, cells were stained with Propidium Iodide (PI) (1 µg/mL) in order to assess cell viability (always higher than 90% after the different treatments). Nonspecific fluorescence was assessed by using an isotype-matched irrelevant mAb (R&D System, Minneapolis, USA) followed by the same secondary Ab. Patient-derived plasma cells (2x10^6^ cells/mL) were cultured for 48h in BMSC-CM or complete medium supplemented with IL-3 (20 ng/mL) and IL-6 (2 ng/mL). The membrane expression of MICA was analyzed by immunofluorescence staining with anti-MICA/AF488 or matched isotype control. All samples were also stained with Fixable Viability Stain 450 (FVS450) (BD Biosciences, San Jose, California, USA) to discriminate cell viability.

NK cell-mediated degranulation was evaluated using the lysosomal marker CD107a as previously described (32, 33). As source of effector cells, we used PBMCs purified from healthy donor blood by Ficoll–Hypaque centrifugation or freshly cultured NK cells. SKO-007(J3) cells were incubated with PBMCs or with NK cells at effector:target (E:T) ratio of 1:1 and 2.5:1 respectively, in a U-bottom 96-well tissue culture plate in complete medium at 37°C and 5% CO2 for 2h. When PBMCs were used, cells were stained with Fixable Viability Stain APC-H7, anti-CD3/APC-H7, anti-CD19/APC-H7, anti-CD14/APC-H7, anti-CD45/Pecy7, anti-CD56/BV421 and anti-CD107a/APC. When cultured NK cells were used, cells were stained with Fixable Viability Stain APC-H7, anti-CD3/APC-H7, anti-CD56/BV421 and anti-CD107a/APC. In some experiments, cells were pre-treated for 20 min at room temperature with anti-NKG2D neutralizing mAb. Fluorescence was analyzed using a FACSCanto or FACS LRSFORTESSA flow cytometer (BD Biosciences, San Jose, California, USA) and data were analyzed by FlowJo V10 Cytometric Analysis Software (BD Biosciences, San Jose, California, USA).

### Western-blot analysis

For Western-Blot analysis, whole cell extracts were obtained from SKO-007(J3) cells as previously described (34). Protein concentration was determined by the BCA method (Thermo Fisher Scientific, Waltham, MA USA). Thirty to 50 μg was resolved by SDSPAGE and transferred to nitrocellulose membranes (Whatman GmbH, Dassel, Germany). After blocking in bovine serum albumin, membranes were probed with specific Abs. An HRP-conjugated secondary Ab and an ECL detection system (Amersham, GE Healthcare, Wisconsin, MA, USA) were used to reveal immunoreactivity.

### Plasmids

The pGL3 Basic -270bp MICA and −270bp MICA/Luc DEL 1 promoter constructs have been already described in (10). The lentiviral vector pHAGE-3xNF-kB-LUC-GFP expressing the green fluorescence gene insert and containing the luciferase gene driven by NF-kB-responsive consensus sequences and the plasmids pVSG-5, pPAX2, pGAg-Pol-Env, and pTK-Green Renilla (TK Renyl) were purchased from Addgene (Cambridge, Massachusetts, USA). The lentiviral vectors pLKO.1sh-GAS6, pLKO.1sh-AXL and pLKO.1sh-PROS1 expressing a short hairpin RNA for GAS6, AXL and PROS1 respectively, and the puromycin gene resistance, were generated by inserting shRNA sequences in pLKO.1 lentiviral vector purchased from Addgene (Cambridge, Massachusetts, USA). The following forward and reverse shRNA sequences were used: human GAS6 shRNA forward, 5’CCGGGCAGACAATCTCTGTTGAGGACTCGAGTCCTCAACAGAGATTGTCTGCTTTTTG-3’; human GAS6 shRNA reverse: 5’-AATTCAAAAAGCAGACAACTCTGTTGAGGACTCGAGTCCTCAACAGAGATTGTCTGC-3’; human AXL shRNA forward: 5’-CCGGCGAAATCCTCTATGTCAACATCTCGAGATGTTGACATAGAGGATTTCGTTTTTG-3’; human AXL shRNA reverse: 5’-AATTCAAAAACGAAATCCTCTATGTCAACATCTCGAGATGTTGACATAGAGGATT TCG-3’; human PROS1 shRNA forward, 5’ CCGG CCTACAAATGACAGTTTCAAT CTCGAG ATTGAAACTGTCATTTGTAGGTTTTTG -3’; human PROS1 shRNA reverse: 5’- AATTCAAAAA CCTACAAATGACAGTTTCAAT CTCGAGATTGAAACTGTCATTTGTAGG-3’. All constructs were verified by DNA sequence analysis. For knocking down MERTK, we used pLKO.1-shMERTK (TRCN0000442967) lentiviral vector and the control vector pLKO.1 non-targeting shRNA (MISSION™ Sigma-Aldrich, St. Louis, MO, USA).

### DNA transfections, virus production and *in vitro* transduction

For virus production, HEK293T was transfected with viral DNA together with packaging vectors, using Lipofectamine 2000 (Life Technologies, Gaithersburg, MD) as previously described (35). SKO-007(J3) and BMSCs cells were infected as previously described (17). SKO-007(J3) cell stable clones expressing pHAGE-3xNF-kB-LUC-GFP were previously described (17). For GAS6, PROS1, AXL and MERTK silencing, MM cell lines were allowed to expand for 24 h and were then selected for 3 days for puromycin resistance (1 µg/mL). SKO-007(J3) cells were transfected using Amaxa nucleofection procedure (Lonza Bioscience, Morrisville, USA) as already described (17) and treated with BMSC-conditioned medium. After 48h, cells were collected, and protein extracts were prepared for the luciferase assay. A TK-renilla expression vector was co-transfected each time to normalize DNA uptake. Luciferase and renilla activity were read using Dual-Luciferase Reporter Assay and the Glomax Multi Detection System (Promega, Madison, USA) following the manufacturer’s instructions.

### RNA isolation and quantitative real-time polymerase chain reaction (qRT-PCR)

Total RNA was extracted using the total RNA mini-Kit following instructions provided by the manufacturer (Geneaid Biotech Ltd, Taiwan) and 2 µg were used for cDNA first-strand synthesis in a 25 µl reaction volume according to the manufacturer’s protocol for M-MLV reverse transcriptase (Promega, Madison, USA). MICA, GAS6 and GAPDH mRNA expression were analyzed by real-time PCR using the following specific TaqMan Gene Expression Assays (Applied Biosystems, Foster City, CA): MICA (Hs00792195_m1), GAS6 (Hs1090305_m1), PROS1 (Hs00165590_m1) and GAPDH (Hs03929097_g1) conjugated with fluorochrome FAM.

The level of expression was measured using Ct (threshold cycle). Relative expression of each gene versus the housekeeping gene was calculated according to the 2^-ΔΔCt^ method. The analysis was performed using the SDS version 2.4 software (Applied Biosystems, Foster City, CA).

### Annexin V

Apoptotic cell death was evaluated using APC Annexin-V Apoptosis Detection Kit with PI (Thermo Fisher Scientific, Waltham, MA USA). Briefly, 1.5 × 10^4^/mL SKO-007(J3) cells infected with lentivirus pLKO.1-shRNA-GAS6 or non-target shRNA were culture in 24-well plates for 72h. Cells were then stained using Annexin-V/APC and PI according to the manufacturer’s instruction. Cell populations were acquired using FACS Canto II flow cytometer (BD Biosciences, San Jose, California, USA). Flow cytometric analysis was performed using Flow Jo Flow Cytometric Analysis Software.

### Enzyme-linked immunosorbent assay (ELISA)

BMSC-conditioned medium and bone marrow plasma were analyzed for GAS6 by ELISA following instructions provided by the manufacturer (R&D System, Minneapolis, USA). BMSC-CM was collected from 72h culture of 20x10^3^ BMSCs in 1 mL of serum free-medium in a 24-well plate. Absorbance was measured at 450/540nm with Victor2 Microplate Reader (Perkin Elmer – GMI Waltham, Massachusetts, USA), and GAS6 concentration was calculated in correlation to a standard curve of control samples.

### Preparation of BMSC-CM

Medium collected from 72h-culture of confluent BMSCs (2x10^4^ cells) (BMSCs-CM) was used to stimulate MM cell lines for 72h. In some experiments, BMSCs-CM was treated with Proteinase K (250µg/mL) (Sigma-Aldrich, St. Louis, MO, USA) for 1h at 65°C. BMSCs-CM and complete RPMI1640 medium were separated in different fractions by Amicon Ultra-15 centrifugal filter devices (Merck Millipore, Darmstadt, Germany) according to the manufacturer’s instructions.

### Statistical analysis

Statistical significance between two groups was determined by performing two-tailed, Student’s t-test, Anova. Prism 6 (GraphPad) software was used. Graphs show mean values, and error bars represent the SD or SEM.We used non-parametric t-test (Mann Whitney test). P-value of < 0.05 (*), < 0.01 (**), < 0.001 (***), and < 0.0001 (****) was considered statistically significant, when not indicated, data were not statistically significant. Multiple comparisons were performed using univariate analysis of variance (Two-way Anova with Bonferroni’s post-test).

## Results

### GAS6/TAM signaling pathway regulates MICA expression in human MM cells

GAS6 and its receptors TYRO3, AXL and MERTK contribute to MM pathobiology (21, 22), however the role of these molecules in the regulation of immune attack of malignant PCs remains to be elucidated.

**Figure 1:**
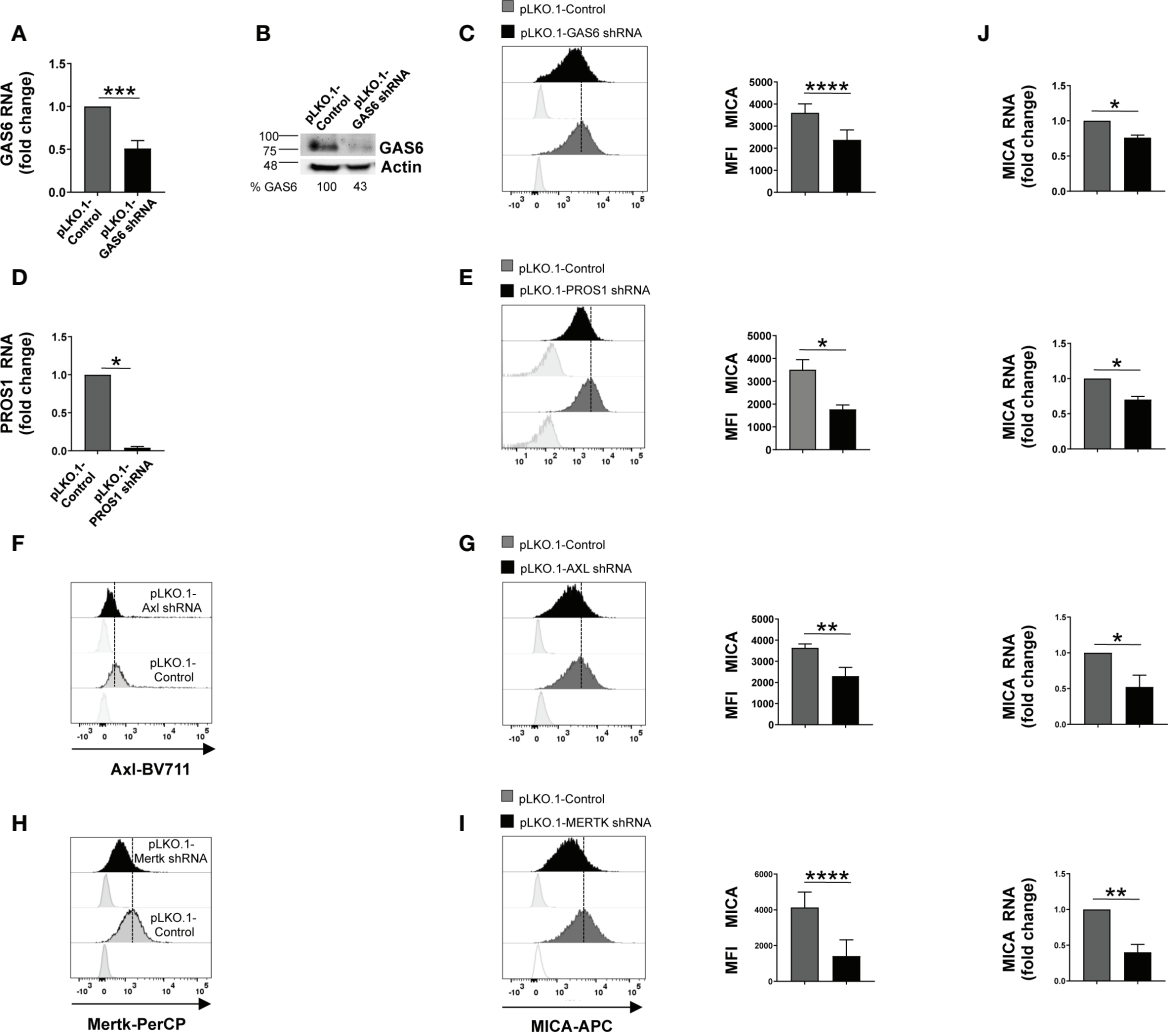
Silencing of GAS6, PROS1 or TAM receptors reduces MICA expression in SKO-007(J3) cells. Total mRNA and protein extracts obtained from SKO-007(J3) pLKO.1-GAS6 shRNA or pLKO.1-PROS1 shRNA and pLKO.1-Control were analyzed for GAS6 or PROS1 expression by real-time PCR **(A–D)** and Western Blot **(B)**. AXL and MERTK receptor expression was analyzed by FACS on SKO-007(J3) pLKO.1-Control, pLKO.1-AXL shRNA **(F)** or pLKO.1-MERTK shRNA **(H)**. MICA expression was analyzed by flow cytometry and real-time PCR in SKO-007(J3) pLKO.1-GAS6 shRNA (n=6) **(C)**, pLKO.1-PROS1 shRNA (n=3) **(E)**, pLKO.1-AXL shRNA (n=6) **(G)**, pLKO.1-MERTK shRNA (n=6) **(I)** or pLKO.1-Control. Histograms represent the MFI of specific mAb subtracted of MFI of isotype control. Data are shown as mean ± SD (****p < 0.001; ***p < 0.005; **p < 0.01; *p < 0.05 Mann-Whitney Test). For real-Time PCR analysis **(J)**, data, expressed as fold change units, were normalized with GAPDH and referred to pLKO.1-Control, considered as calibrator. Data are shown as mean ± SD (**p < 0.01; *p < 0.05; Mann-Whitney Test).

Here, we investigated the effect of GAS6/TAM signaling pathway on the regulation of NKG2D ligands in three MM cell lines [SKO-007(J3), U266 and ARP1]. Among TAM receptors, these MM cell lines lack TYRO3 but express MERTK, while AXL is present only on SKO-007(J3) cells (**Supplementary Figures 1A**, **2A–E**). Thus, we performed a flow cytometric analysis on MM cells silenced for the ligands GAS6 and PROS1 or the receptors AXL and/or MERTK by lentiviral-transduced small-hairpin RNAs (shRNAs). Since stable depletion of these proteins is cytotoxic for MM cells (20, 22), these experiments were performed using transient infection without any significant effect on cell viability (as assessed by Annexin V staining) (**Supplementary Figure 1C**). We found that knockdown of GAS6, PROS1, AXL or MERTK was able to significantly reduce MICA surface levels in MM cell lines (**Figures 1C, E, G, I** and **Supplementary Figures 2B, C, F, G**). Differently, depletion of GAS6 did not affect the expression of other NKG2D ligands (MICB and ULBPs), NKp30 ligand B7/H6, DNAM-1 ligand PVR, MHC class I and of the adhesion molecule ICAM-1 (**Supplementary Figure 1B**).

These results indicate a role for TAM pathway in sustaining the basal expression of MICA in MM cells.

We then examined whether MICA downregulation could be the consequence of a decreased mRNA expression. To this aim, total RNA was isolated from SKO-007(J3) cells infected with lentivirus pLKO.1-shRNA GAS6, PROS1, AXL or MERTK or non-target shRNA, and analyzed by real-time quantitative RT-PCR. Consistent with FACS analysis, silencing of these proteins decreased basal *MICA* mRNA levels (**Figure 1J**).

**Figure 2:**
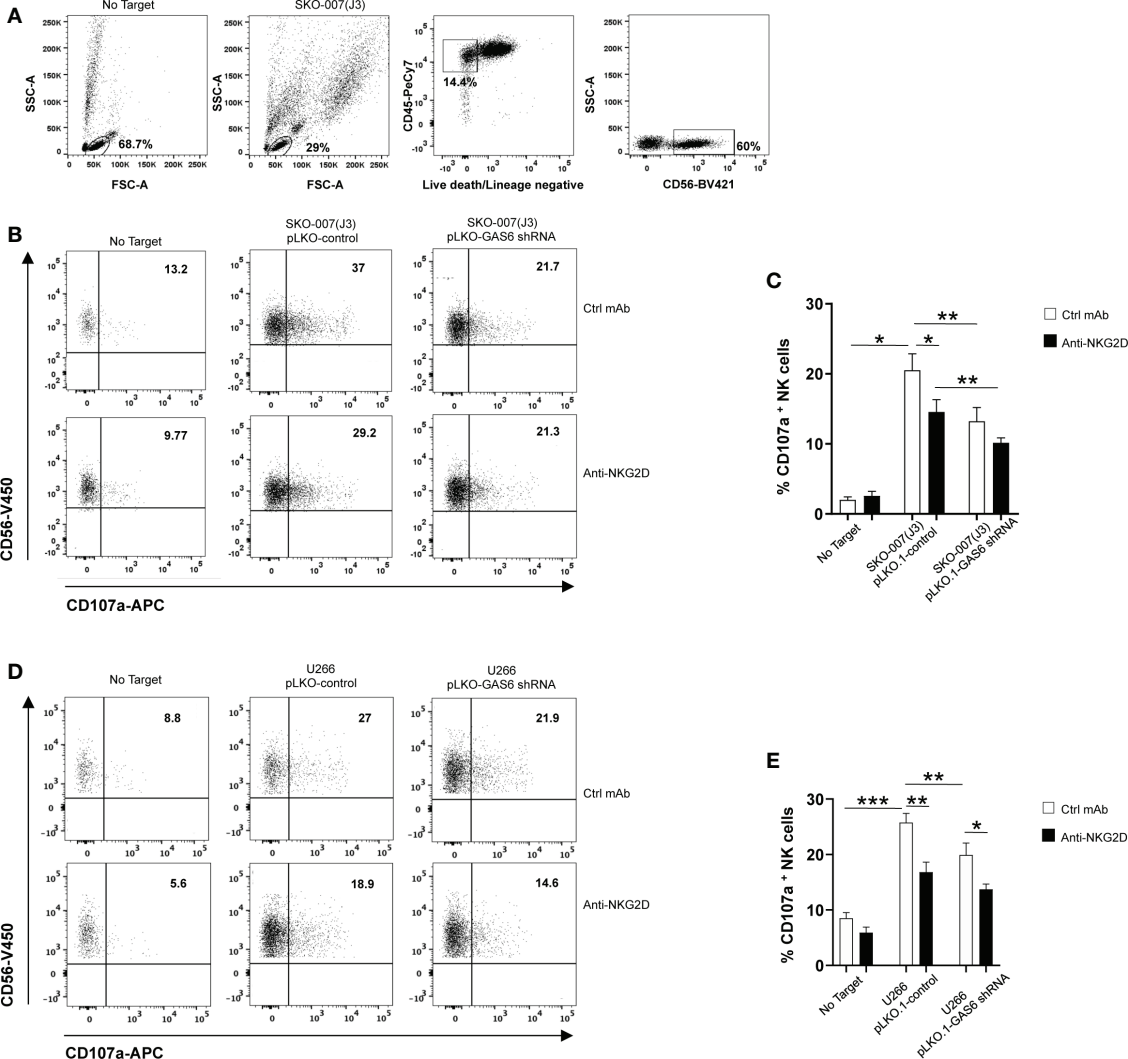
Impaired NKG2D-dependent recognition of GAS6-depleted MM cells. Healthy donor-derived PBMCs were incubated with SKO-007(J3) **(A–C)** or U266 **(D, E)** pLKO.1-GAS6 shRNA or pLKO.1-Control at E/T ratio of 1:1. CD107a expression was analyzed on CD3^-^CD19^-^CD14^-^CD45^+^CD56^+^ NK cells as shown in **(A, B)**. To evaluate the role of NKG2D, the assay was performed in parallel treating NK cells with blocking anti-NKG2D or anti-IgG mAb used as control (Ctrl). Results are expressed as the percentage of CD107a^+^ NK cells. A representative experiment for each MM cell line is shown in **(B, D)**. Histogram represents the mean ± SD from three independent experiments **(C, E)** (*p < 0.05; **p < 0.001 ; ***p < 0.005 ANOVA).

To evaluate the functional consequences of changes in MICA expression, we analyzed the degranulation of healthy donor NK cells against SKO-007(J3) cells or U266 infected with lentivirus pLKO.1-shRNA GAS6 or non-target shRNA (pLKO.1-control). As shown in **Figures 2B–E**, expression of CD107a on NK cells was decreased when co-cultured with GAS6 depleted-SKO-007(J3) or U266 target cells. Moreover, a blocking anti-NKG2D mAb impaired NK cell degranulation against SKO-007(J3) or U266 pLKO.1-control, and U266 pLKO.1-GAS6 shRNA; however, it failed to reduce the activation of NK cells contacting SKO-007(J3) pLKO.1-GAS6 shRNA.

In lines with previous studies (6, 10, 11, 36), these findings demonstrate that constitutive NK cell degranulation against MM cells involves NKG2D. More importantly, they indicate that MICA downregulation on GAS6 depleted- SKO-007(J3) and U266 cells compromises their NKG2D-dependent recognition by NK cells.

### BMSC-derived GAS6 increases MICA surface expression in MM cells

**Figure 3:**
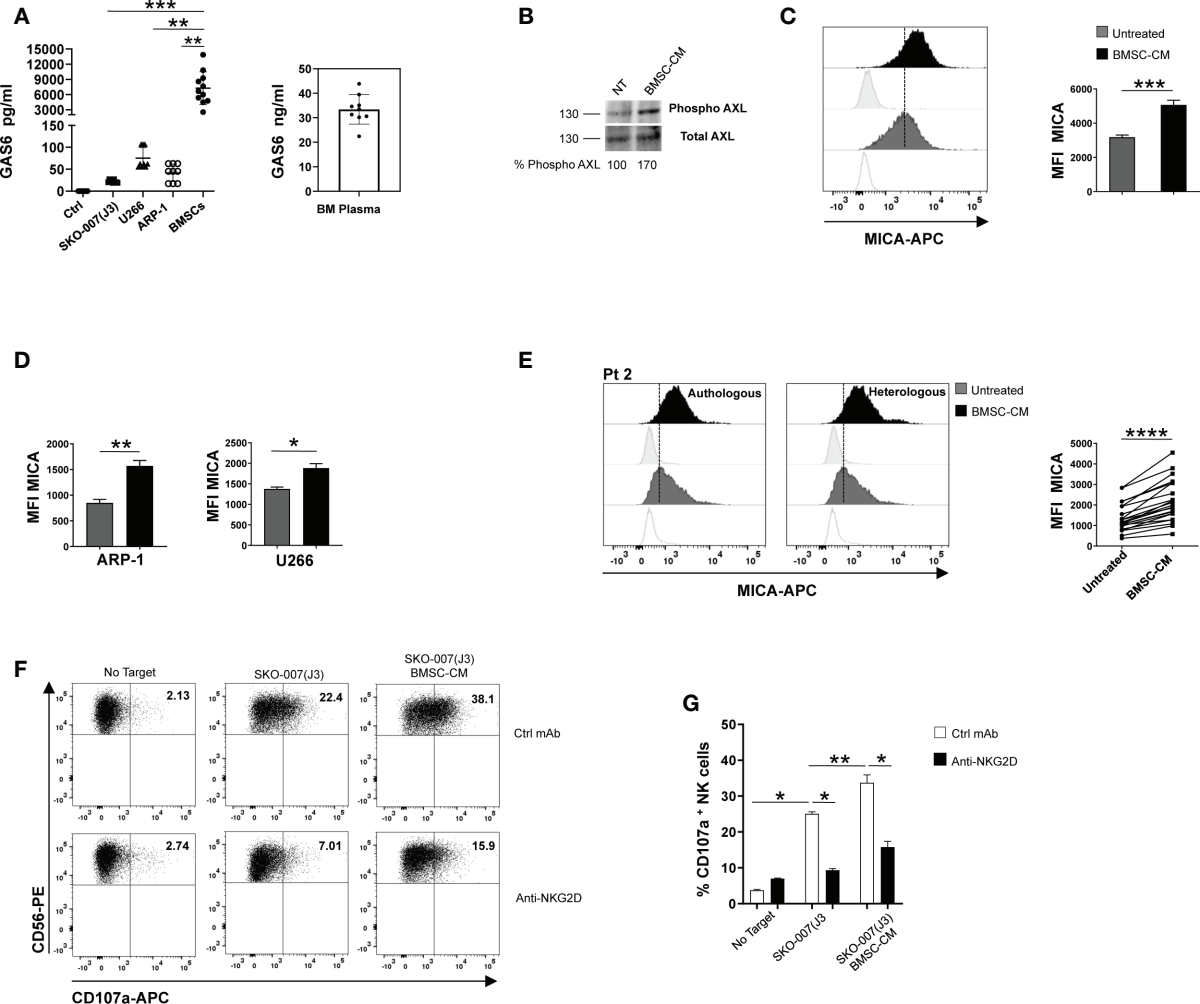
BMSC-CM up-regulates MICA surface expression in MM cells. GAS6 protein was quantified by ELISA in conditioned medium (CM) derived from the indicated MM cell lines, BMSCs and plasma from BM aspirates of MM patients. Serum free medium was used as control (Ctrl) **(A)**. Total protein extracts obtained from SKO-007(J3) untreated or treated for 1h with BMSC-CM were analyzed for phosphorylated and total AXL expression by Western Blot **(B)**. Numbers represent densitometric analysis of phosphorylated AXL normalized relative to untreated cells. MICA surface expression was analyzed by flow cytometry in SKO-007(J3) (n=8) **(C)**, ARP-1 (n=6) or U266 (n=5) MM cell line **(D)** or in primary malignant PCs (n=12) **(E)** untreated or treated with BMSC-CM for 72h or 48h, respectively. A representative experiment is shown. Histograms represent the MFI of specific mAb subtracted of MFI of isotype control. Data show mean ± SD (****p < 0.001; ***p < 0.005; **p < 0.05; Mann -Whitney Test). NK cells derived from healthy donor PBMCs were incubated with SKO-007(J3) cells, untreated or cultured for 72h with BMSC-CM and used as target cells in a degranulation assay as described above. Results are expressed as the percentage of CD107a^+^ NK cells **(F)**. A representative experiment is shown in **(E)**. Histogram represents the mean ± SD from three independent experiments **(G)** (*p < 0.05; **p < 0.001 ANOVA).

Myeloma BM microenvironment is highly enriched in GAS6 (22), which is also largely produced by BMSCs and tumor cells (**Figure 3A** and **Supplementary Table 1**). We thus analyzed the possible effects of MM patient derived-BMSCs on MICA expression in MM cells. We observed that conditioned medium (BMSCs-CM) collected after 72h culture of MM-BMSCs could induce AXL phosphorylation in SKO-007(J3) (**Figure 3B**) and up-regulated the basal membrane expression of MICA on SKO-007(J3), ARP-1 and U266 cell lines (**Figures 3C, D**). Moreover, we found that MM patient-derived PCs cultured in autologous or heterologous BMSC-CM for 48h expressed higher levels of MICA than primary myeloma cells cultured in RPMI1640 medium alone (**Figure 3E** and **Supplementary Table 2**). However, BMSC-CM did not affect the expression of MICB on these cells (data not shown). Consistently, BMSC-CM-treated SKO-007(J3) were more capable to activate NK cell degranulation. This effect was significantly inhibited by a blocking anti-NKG2D mAb, indicating that stimulation of NK cell degranulation was dependent on NKG2D activation (**Figures 3F, G**).

**Figure 4:**
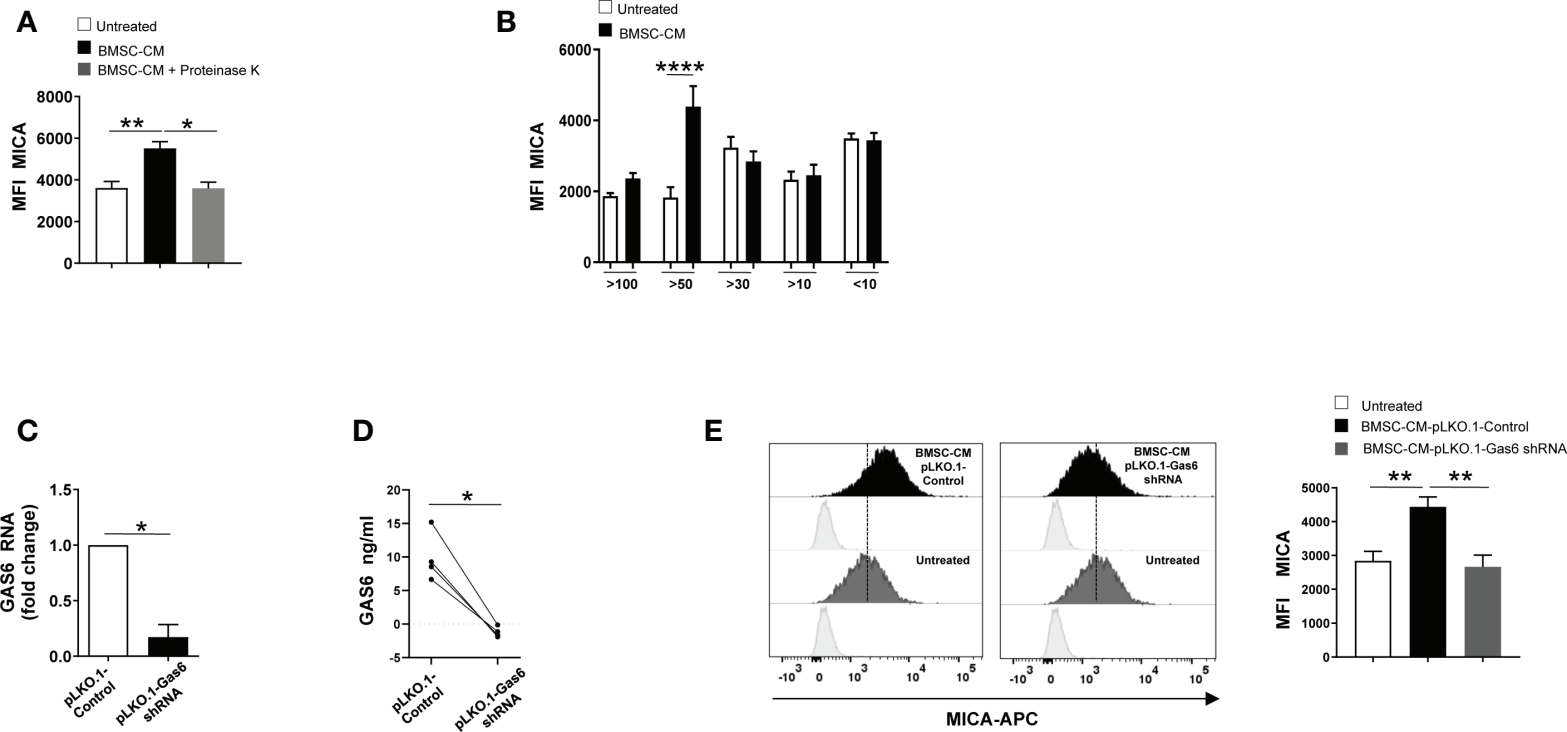
GAS6 controls MICA up-regulation in BMSC-CM-treated MM cells. Flow cytometry analysis of MICA surface expression on SKO-007(J3) cells cultured for 72h in BMSC-CM, pre-treated or not with proteinase K **(A)**, or in fractions of different molecular weight obtained from BMSC-CM (n=8) **(B)**. Histograms represent the MFI of specific mAb subtracted of MFI of isotype control. Data show mean ± SD (*p < 0.05; **p < 0.005; ****p < 0.001; ANOVA). Analysis of *GAS6* mRNA **(C)** and protein **(D)** in BMSCs infected with lentiviral vector expressing GAS6 shRNA (pLKO.1-GAS6 shRNA) or scramble control (pLKO.1-Control) (n=4). Data show mean ± SD (*p < 0.05; Mann-Whitney Test). MICA surface expression was analyzed by FACS on SKO-007(J3) treated for 72h with CM derived from BMSCs pLKO.1-GAS6 shRNA or pLKO.1-Control (n=3) **(E)**. Histograms represent the MFI of specific mAb subtracted of MFI of isotype control. Data show mean ± SEM (**p < 0.001; ANOVA).

To gain insight into the nature of the BMSCs-derived factor(s) capable of enhancing MICA expression on MM cells, BMSC-CM was pre-treated with the serine protease Proteinase K for 1h before to be used to stimulate SKO-007(J3) cells for 72h. As shown in **Figure 4A**, we found that pre-treatment with this enzyme able to degrade proteins abolished the capability of BMSC-CM to increase the expression of MICA on MM cells. These findings indicate that soluble factor(s) produced by BMSCs involved in these mechanisms are protein(s). Then, using centrifugal filtration with a semi permeable membrane of different molecular weight cutoff, we separated BMSC-CM and complete RPMI1640 medium, used as a control, into fractions ranging from 100 to 10 KDa. Fractioned BMSC-CM or complete RPMI1640 medium were used to stimulate SKO-007(J3) cells for 72h. In line with our data implying GAS6 (75 KDa) in MICA up-regulation, we observed that this NKG2D ligand augmented in SKO-007(J3) cells exposed to the fraction >50KDa (**Figure 4B**). Consistently, CM derived from BMSCs lacking GAS6 (**Figures 4C, D**) was not able to augment MICA expression on SKO-007(J3) cells (**Figure 4E**).

Taken together, these results demonstrate that autocrine and paracrine production of GAS6 is implicated in the regulation of MICA on MM cells.

### Role of NF-kB in MICA up-regulation by BMSCs

**Figure 5:**
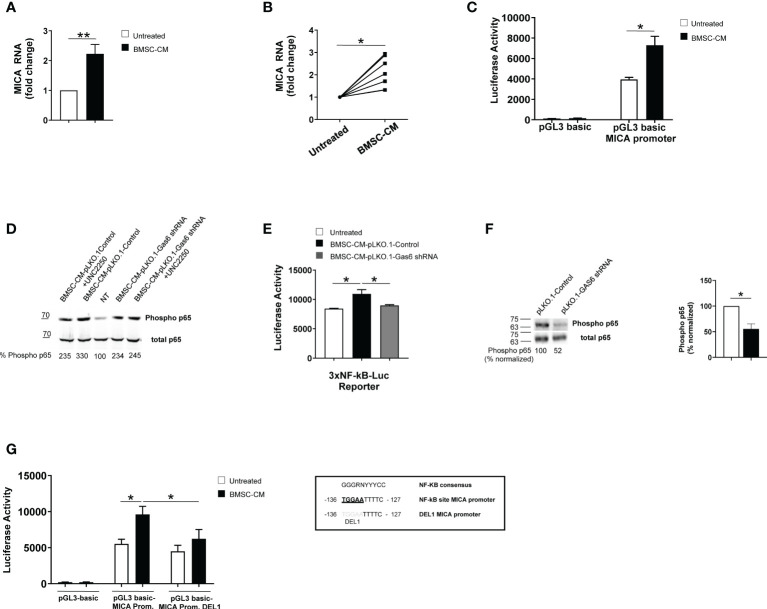
BMSC-CM increases *MICA* mRNA expression and promoter activity *via* NF-kB activation. Real time PCR analysis of total mRNA obtained from SKO-007(J3) cells **(A)** or patient-derived PCs **(B)** after 48h stimulation with BMSC-CM or complete medium. Data, expressed as fold change units, were normalized with GAPDH and referred to the untreated cells, considered as calibrator. For SKO-007(J3) cells, histograms represent the mean ± SD from six independent experiments. For primary PCs, data from 7 MM patients are shown where each dot represents a single patient. (*P< 0.05; **P<0.005; Mann-Whitney Test). SKO-007(J3) cells were transiently transfected with pGL3 basic empty vector/MICA 270bp promoter plasmid **(C)** and/or a DEL1-270bp MICA promoter plasmid (in which a putative NF-kB site indicated in the figure was removed by site-directed mutagenesis) **(G)** or infected with lentivirus pHAGE-3xNF-kB-LUC-GFP **(E)** as described in materials and methods. After 48h of treatment with BMSC-CM, SKO-007(J3) cells were harvested, and protein extracts were prepared for the luciferase assay. Data, expressed as relative luciferase activity, were normalized to protein concentration and renilla activity and represent the mean±± SEM from three independent experiments (*p < 0.05; ANOVA). Total protein extracts obtained from SKO-007(J3) treated with CM derived from BMSCs pLKO.1-GAS6 shRNA or pLKO.1-Control in the absence or in the presence of UCN2250 (1 µM) **(D)** or SKO-007(J3) infected with lentivirus pLKO.1-GAS6 shRNA or pLKO.1-Control **(F)** were analyzed for phosphorylated and total p65 expression by Western Blot. Numbers represent densitometric analysis of phosphorylated p65 normalized relative to untreated cells.

To examine the molecular mechanisms underlying MICA up-regulation, we performed real-time quantitative RT-PCR on total RNA isolated from SKO-007(J3) cells and PCs isolated from different MM patients cultured in BMSC-CM for 48h. We found a significant increase of *MICA* mRNA levels in treated MM cells (**Figures 5A, B**). Thus, we investigated the direct effect of BMSC-CM on the activity of the *MICA* promoter. As shown in **Figure 5C**, by transient transfection assays, we observed that BMSC-CM enhanced the luciferase reporter activity driven by a 270bp 5′-flank of *MICA* promoter.

Collectively, these data indicate that *MICA* mRNA expression and promoter activity are enhanced by BMSC-CM in MM cells.

Previous reports demonstrated a role for NF-kB in the regulation of MICA expression in different types of cells (e.g. T lymphocytes, endothelial cells and MM cells) (37, 38) NF-kB proteins are constitutively active in MM cells but BMSCs produce many soluble factors, including GAS6, which further trigger this signaling pathway in these tumor cells (16, 20, 39, 40). For this reason, we investigated the role for NF-kB in MICA regulation by BMSCs.

First, we observed that GAS6 depleted-BMSC-CM has a reduced capability to induce phosphorylation of p65 and to enhance NF-kB transcriptional activity in SKO-007(J3) cells; moreover, the TAM receptor inhibitor UNC2250 partially blocks phosphorylation of p65 by BMSC-CM in these cells (**Figures 5D, E**). Accordingly, we found reduced levels of phosphorylated p65 in GAS6 silenced SKO-007(J3) cells (**Figure 5F**). These findings indicate a direct contribute of autocrine and paracrine GAS6 as a regulator of NF-kB pathway in MM cells.

Second, we revealed that the stimulatory effect of BMSC-CM was significantly diminished on a mutated version of *MICA* promoter (indicated as DEL1), in which a putative NF-kB site (41) was removed by site-directed mutagenesis (**Figure 5G**), thus indicating that binding of this transcription factor to this region is required for promoter activation.

Taken together, these findings demonstrate that NF-kB can act as an activator of MICA expression in MM cells in response to GAS6 secreted by BMSCs.

## Discussion

Natural Killer cells are important effectors of anti-MM immune response. Yet, malignant PCs acquire the ability to elude NK cell recognition and suppress their function (7, 42). To overcome NK cell dysfunctions associated to disease progression, innovative treatments include the adoptive NK cell therapy and monoclonal antibodies to enhance NK cell-versus-MM effect (8, 9); in parallel, tumor modulating agents able to render MM cells more susceptible to NK cell attack also represent promising therapeutic tools. NKG2D is a key activating receptor involved in these mechanisms (6, 43), thus understanding how expression of its ligands is regulated on malignant PCs could be helpful to address these approaches.

Regulation of NKG2D ligand expression in MM cells mainly relays on transcriptional and post-translational mechanisms (44). A number of transcription factors, highly expressed and active in these tumor cells, where they control aberrant and malignancy-specific gene expression program, are also involved in the regulation of NKG2D ligand expression. Our group identified Hsp90, STAT3, IRF4, C-MYC and IKAROS/AIOLOS as repressors of *MICA* and/or *MICB* gene transcription (10–13). These findings indicate that MM cells have developed the capability to exploit the same pathways to promote their growth as well as to reduce immune recognition. Further, a relevant immune escape mechanism is represented by the down-regulation of NKG2D ligands on the surface of MM cells by proteolytic shedding (45). In particular, soluble MICA is overexpressed in the serum of MM patients, and its levels correlate with tumor progression (46).

However, little is currently known about signaling events and molecular mechanisms responsible for basal expression of NKG2D ligands in MM cells.

Here, we provide first evidence that surface MICA expression is regulated by GAS6/TAM pathway in MM cells. We demonstrate that: (1) GAS6/AXL and MERTK signaling, in a cell-autonomous way, sustains MICA expression in MM cells; (2) GAS6 secreted by BMSCs augments membrane MICA expression on MM cells at transcriptional level; (3) NF-kB pathway mediates MICA up-regulation by GAS6.

GAS6/TAM signaling components are overexpressed in MM cells and support their survival and proliferation (20, 22). We found that MM cells silenced for the ligand GAS6 or PROS1, or the receptors AXL and MERTK by lentiviral-transduced shRNAs express reduced levels of MICA, both at protein and mRNA level, and are less susceptible to NKG2D mediated-recognition by NK cells.

The contribution of soluble factors in the regulation of this ligand has been previously described. A number of cytokines and growth factors, such as TGF-β, IL-10, IFN-γ and EGF function as regulators of MICA expression in different types of cancer cells (47–50).

Here, we demonstrate that a peculiar regulatory circuitry, involving both autocrine and paracrine GAS6, assures MICA expression on MM cells. Indeed, MM BM microenvironment is highly enriched in GAS6 (22), which is largely produced by BMSCs. We found a significant up-regulation of membrane MICA expression on MM cell lines as well as on patient-derived PCs by BMSC-CM. However, knockdown of GAS6 in BMSCs abolished these effects. Interestingly, we have already described that BMSCs enhance PVR surface expression on MM cells and promote their NK cell-mediated recognition (17). Our novel findings confirm that expression of NK cell activating ligands on malignant PCs is highly dependent on BM microenvironment.

Our data also indicate that the transcription factor NF-kB is critically involved in MICA regulation by GAS6/BMSCs.

NF-kB was found to be a positive regulator of *MICA* gene in different cellular contexts. In activated T lymphocytes and epithelial cancer cell lines, NF-kB acts by binding to a specific sequence in the long intron 1 of the *MICA* gene (37, 38). Furthermore, in human endothelial cells TNF-α-induced NF-kB binds a regulatory control site at -130 bp upstream of the *MICA* transcription start site (41).

Here, we first observed reduced NF-kB activation in MM cells lacking GAS6 and after treatment with GAS6 depleted-BMSC-CM, thus revealing that TAM receptor signaling controls NF-kB activity in these tumor cells. Second, by a site-directed mutagenesis approach, we proved that deletion of an NF-kB responsive element (41) compromises the induction of *MICA* promoter by BMSC-CM. Based on these observations, we propose a role for NF-kB as a direct positive regulator of *MICA* promoter in MM cells.

Previous studies have described a negative impact of TAM pathway on immunological visibility of tumor cells *via* upregulation of NK cell inhibitory ligands. As an immune evasion mechanism, AXL signaling was shown to increase surface expression of MHC-class I molecules and PDL-1 in lung, renal and breast cancer cell lines (51–53). In our experimental setting, either silencing of GAS6 or culture with BMSC-CM did not affect MHC-class I expression in MM cells, while the possible effects on other NK cell inhibitory ligands were not investigated. However, the absence of GAS6 in MM cells reduces their capability to activate NK cells, whereas BMSC-CM renders MM more susceptible to recognition by both NK cells from healthy donors and MM patients (17). Such functional consequences do not suggest any relevant change of inhibitory pathways by GAS6/TAM signaling in MM cells, but further studies are needed to address this possibility.

Although unexcepted, our data are not the first demonstration on the positive regulation of MICA by an oncogenic pathway. Another major cell-autonomous component of tumor progression, EGFR, was shown to drive basal expression of NKG2D ligands in epithelial cancer cells (50). Together these and our observations indicate that oncogenic signaling pathways may be relevant at the early stages of cancer when they become dysregulated, and expression of activating ligands is key for immune surveillance.

Remarkably, GAS6/TAM pathway is also a key regulator of immune homeostasis (25). Innate and adaptive immune cells, in humans and mice, express TAM receptor/ligands and blocking TAM signaling causes severe defects in the clearance of apoptotic cells, widespread inflammation and over-activation of the immune system, and development of systemic autoimmunity (54).

This axis signaling is also known to regulate NK cell differentiation and function. Binding of GAS6 to TAM receptors inhibits murine NK cell function by phosphorylating Cbl-b, which promotes the degradation of LAT1. Consistently, administration of GAS6 or TAM receptor inhibitors leads to rejection of metastatic tumors (28, 29). However, to our knowledge, there is no evidence of these inhibitory effects on human NK cells. In contrast, a recent study proposed TYRO3 as an NK cell activating receptor showing that activated human NK cells can acquire the receptor upon contact with tumor cells *via* trogocytosis and that TYRO3^+^ NK cells are more cytotoxic and produce more IFN-γ (30). Activating effects were also reported for MERTK in human CD8^+^ T cells, where the receptor functions as a co-stimulatory signal promoting proliferation and generation of memory T cells (55).

The role of TAM receptors in the regulation of NK cells in MM has never been investigated. In the context of a study on NK cells from newly diagnosed and refractory relapsed MM, an RNA seq analysis showed a reduction of *AXL* and *GAS6* gene expression (56), thus suggesting that inhibitory signals generated by GAS6/TAM components may not be related to NK cell dysregulation in this malignancy. However, these mechanisms remain to be studied.

Due to their pro-oncogenic and putative immune-inhibitory effects, TAM receptors have emerged as promising targets for cancer therapy. Indeed, many clinical trials are currently investigating TAM targeting therapies in solid and hematologic cancers. However, further investigations are required to elucidate the contribute of different TAM receptors on distinct immune cell types during anti-tumor immune response. Indeed, the impact on immune system and immune surveillance are relevant aspects to evaluate in design drugs targeting cancer cells. In this context, our data suggest that the modulation of MICA on tumor cells may be a potential limiting effect of GAS6/TAM inhibitors on immune surveillance. To this regard, a recent report demonstrated the feasibility of using autologous NK cells bearing NKG2D-CAR to treat MM. These cells have robust cytotoxic activity against MM cells *in vitro* and exhibit high efficiency *in vivo* in a mouse model of MM (57). In this therapeutic perspective, it would be important to preserve and/or enhance NKG2D ligand expression on tumor cells, and our finding could be helpful in setting the right combined therapy.

Overall, these data reveal a novel immunoregulatory role for the GAS6/TAM pathway and shed light on molecular mechanisms underlying basal MICA expression in MM cells.

## Data availability statement

The original contributions presented in the study are included in the article/**Supplementary Material**. Further inquiries can be directed to the corresponding authors.

## Ethics statement

The studies involving human participants were reviewed and approved by RIF.CE: 5191. The patients/participants provided their written informed consent to participate in this study.

## Author contributions

CF, AK, AM, LL, and HS performed the experiments and analyzed the results; HS, ASo, AZ, and MC contributed with analytic tools and analyzed the results; MP and FF provided and managed bone marrow samples from patients; AG and ASa critically reviewed the manuscript. CF and HS contributed to design research and write the manuscript. All authors contributed to the article and approved the submitted version.

## Funding

This work was supported by grants from andMinistero dell’Istruzione, dell’Università e della Ricerca (PRIN cod. 20174T7NXL and 2017NTK4HY), Ricerca Universitaria (RP120172A7CD4ACB).

## Acknowledgments

The authors thank all patients who contributed to this study.

## Conflict of interest

The authors declare that the research was conducted in the absence of any commercial or financial relationships that could be construed as a potential conflict of interest.

## Publisher’s note

All claims expressed in this article are solely those of the authors and do not necessarily represent those of their affiliated organizations, or those of the publisher, the editors and the reviewers. Any product that may be evaluated in this article, or claim that may be made by its manufacturer, is not guaranteed or endorsed by the publisher.

## Supplementary material

Supplementary Figure 1 Defective TAM signaling causes a selective reduction of MICA expression in SKO-007(J3). TAM (TYRO3, AXL, MERTK) receptor expression was analyzed by FACS on SKO-007(J3) cells **(A).** A representative experiment is shown. Flow cytometry analysis of the indicated surface molecules on SKO-007(J3) pLKO.1-GAS6 shRNA or pLKO.1-Control (n=3). A representative experiment is shown (**B**, left panel). Histograms represent the MFI of specific mAb subtracted of MFI of isotype control. Data are shown as mean ± SD (*P< 0.05; Mann-Whitney) (**B**, right panel). SKO-007(J3) pLKO.1-GAS6 shRNA or pLKO.1-Control were stained using Annexin-V/APC and Propidium Iodide. A representative experiment is shown (**C,** left panel). Histograms indicate the percentage of Annexin V or propidium positive cells and were calculated based on at least three independent experiments (**C**, right panel).

Supplementary Figure 2 Depletion of GAS6 or TAM receptors reduces MICA expression in U266 and ARP-1 cells. TAM (TYRO3, AXL, MERTK) receptor expression was analyzed by FACS on U266 **(A)** and ARP-1 cells **(E)**. A representative experiment is shown. MERTK and MICA surface expression was analyzed by FACS on U266 **(B)** or ARP-1 **(F)** pLKO.1-Control and pLKO.1-MERTK shRNA or pLKO.1-GAS6 shRNA **(C, F)** (n=3). In pLKO.1-GAS6 shRNA transduced U266 **(D)** or ARP-1 **(H)** ULBPs, B7/H6, PVR and ICAM1 expression was also analyzed. Histograms represent the MFI of specific mAb subtracted of MFI of isotype control. Data are shown as mean ± SD (*P< 0.05; Mann-Whitney). Total mRNA obtained from U266 **(C)** or ARP-1 (**G)** pLKO.1-GAS6 shRNA or pLKO.1-Control were analyzed for *GAS6* expression by real-time PCR (n=3). Data, expressed as fold change units, were normalized with GAPDH and referred to pLKO.1-Control, considered as calibrator. Data are shown as mean ± SD (**p* < 0.05; Mann-Whitney Test).

Supplementary Table 1 Clinical parameters of MM patients used for the analysis of GAS6 in condition medium derived from BMSCs and BM plasma. Patients were classified according to Durie and Salmon’s Staging System.

Supplementary Table 2 Clinical parameters of MM patients used for the analysis of MICA surface expression and mRNA. Patients were classified according to Durie and Salmon’s Staging System.
