# Supplementary figures and images for: GAS6/TAM signaling pathway controls MICA expression in multiple myeloma cells
Source: Front Immunol. 2022 Jul 28;13:942640. doi: 10.3389/fimmu.2022.942640 (PMC9368199; doi:10.3389/fimmu.2022.942640)

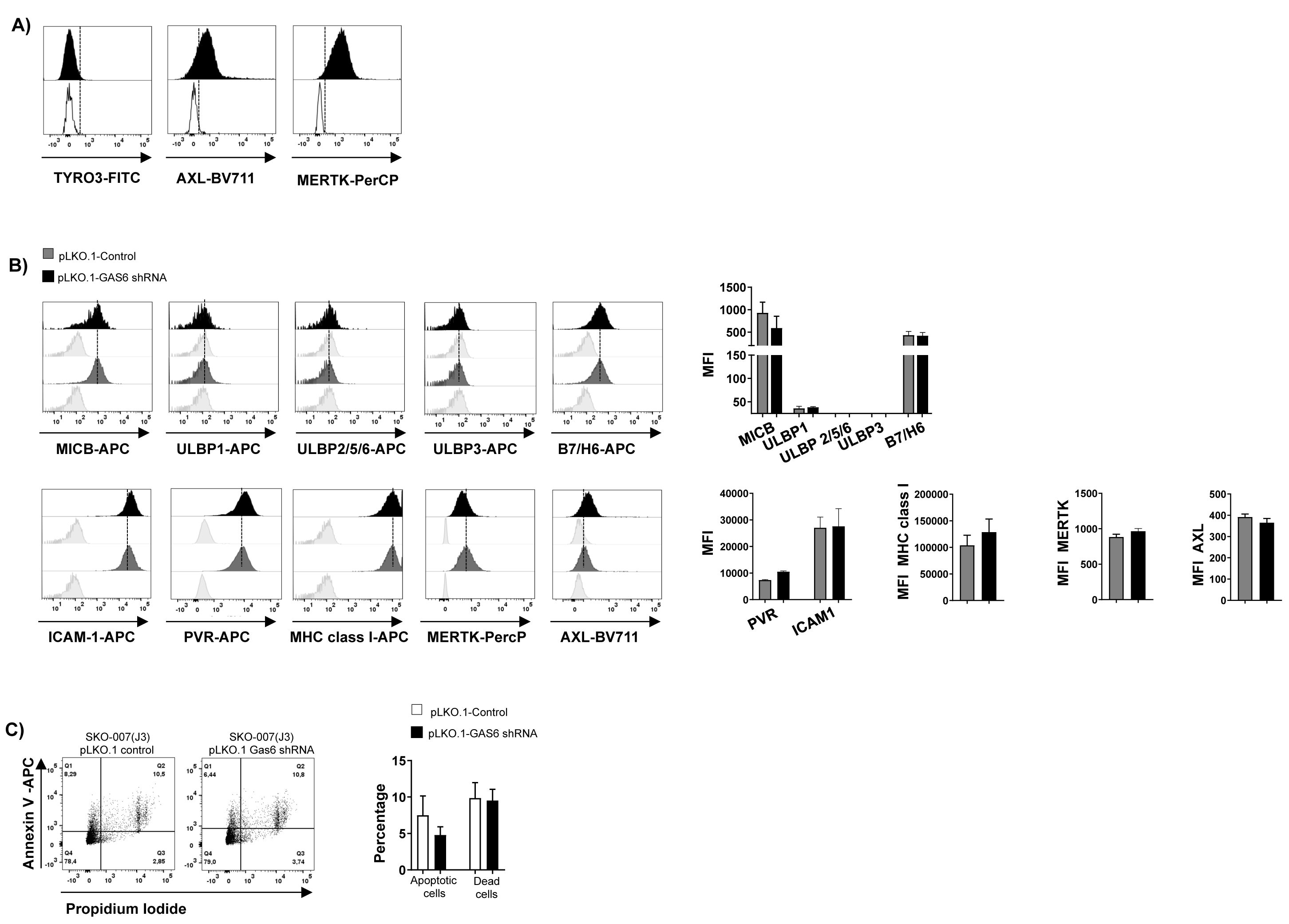

Supplement: Supplementary Figure 1 — Defective TAM signaling causes a selective reduction of MICA expression in SKO-007(J3). TAM (TYRO3, AXL, MERTK) receptor expression was analyzed by FACS on SKO-007(J3) cells (A). A representative experiment is shown. Flow cytometry analysis of the indicated surface molecules on SKO-007(J3) pLKO.1-GAS6 shRNA or pLKO.1-Control (n=3). A representative experiment is shown (B, left panel). Histograms represent the MFI of specific mAb subtracted of MFI of isotype control. Data are shown as mean ± SD (*P< 0.05; Mann-Whitney) (B, right panel). SKO-007(J3) pLKO.1-GAS6 shRNA or pLKO.1-Control were stained using Annexin-V/APC and Propidium Iodide. A representative experiment is shown (C, left panel). Histograms indicate the percentage of Annexin V or propidium positive cells and were calculated based on at least three independent experiments (C, right panel). [file Image_1.tif]

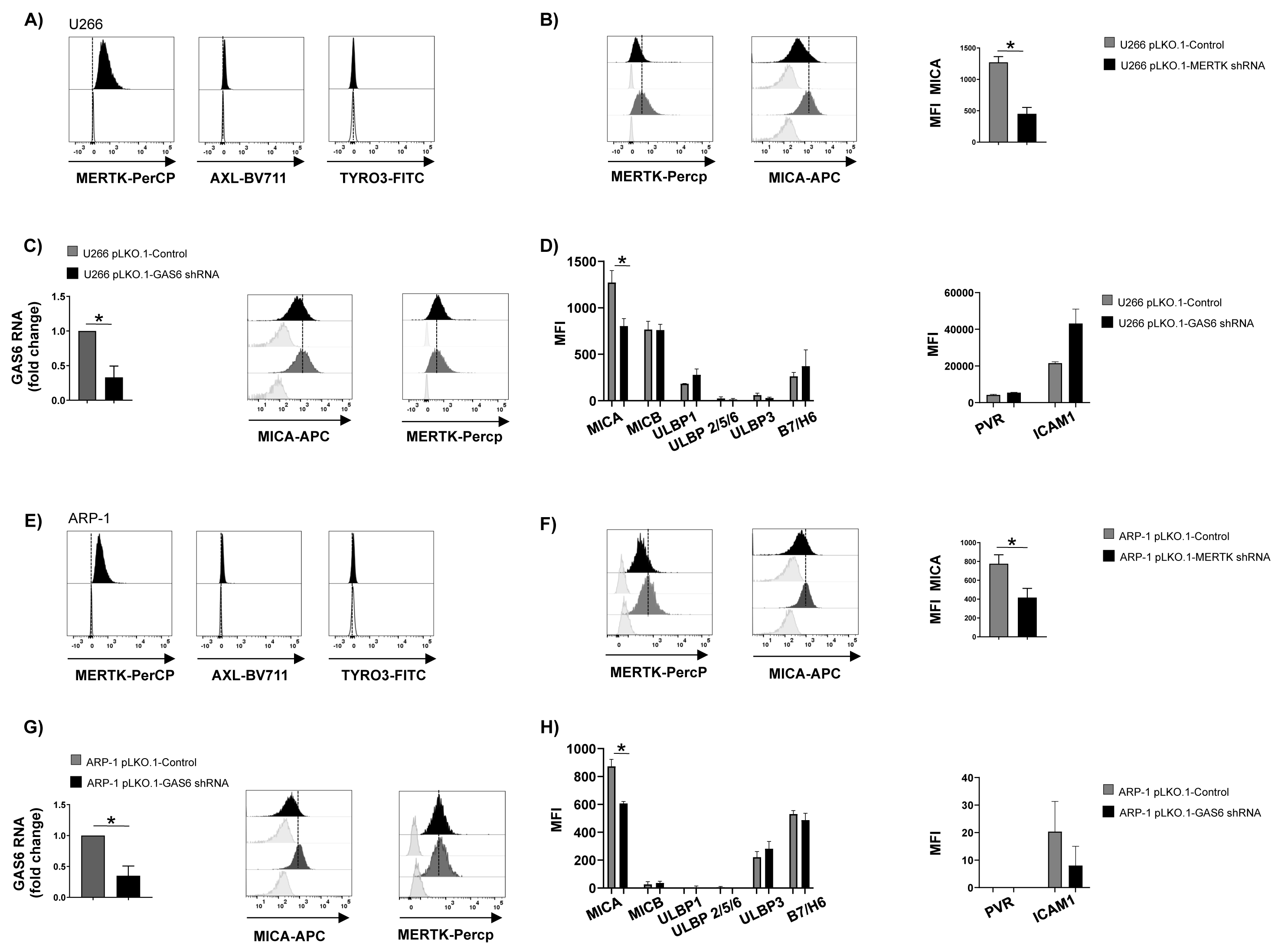

Supplement: Supplementary Figure 2 — Depletion of GAS6 or TAM receptors reduces MICA expression in U266 and ARP-1 cells. TAM (TYRO3, AXL, MERTK) receptor expression was analyzed by FACS on U266 (A) and ARP-1 cells (E). A representative experiment is shown. MERTK and MICA surface expression was analyzed by FACS on U266 (B) or ARP-1 (F) pLKO.1-Control and pLKO.1-MERTK shRNA or pLKO.1-GAS6 shRNA (C, F) (n=3). In pLKO.1-GAS6 shRNA transduced U266 (D) or ARP-1 (H) ULBPs, B7/H6, PVR and ICAM1 expression was also analyzed. Histograms represent the MFI of specific mAb subtracted of MFI of isotype control. Data are shown as mean ± SD (*P< 0.05; Mann-Whitney). Total mRNA obtained from U266 (C) or ARP-1 (G) pLKO.1-GAS6 shRNA or pLKO.1-Control were analyzed for GAS6 expression by real-time PCR (n=3). Data, expressed as fold change units, were normalized with GAPDH and referred to pLKO.1-Control, considered as calibrator. Data are shown as mean ± SD (*p < 0.05; Mann-Whitney Test). [file Image_2.tif]

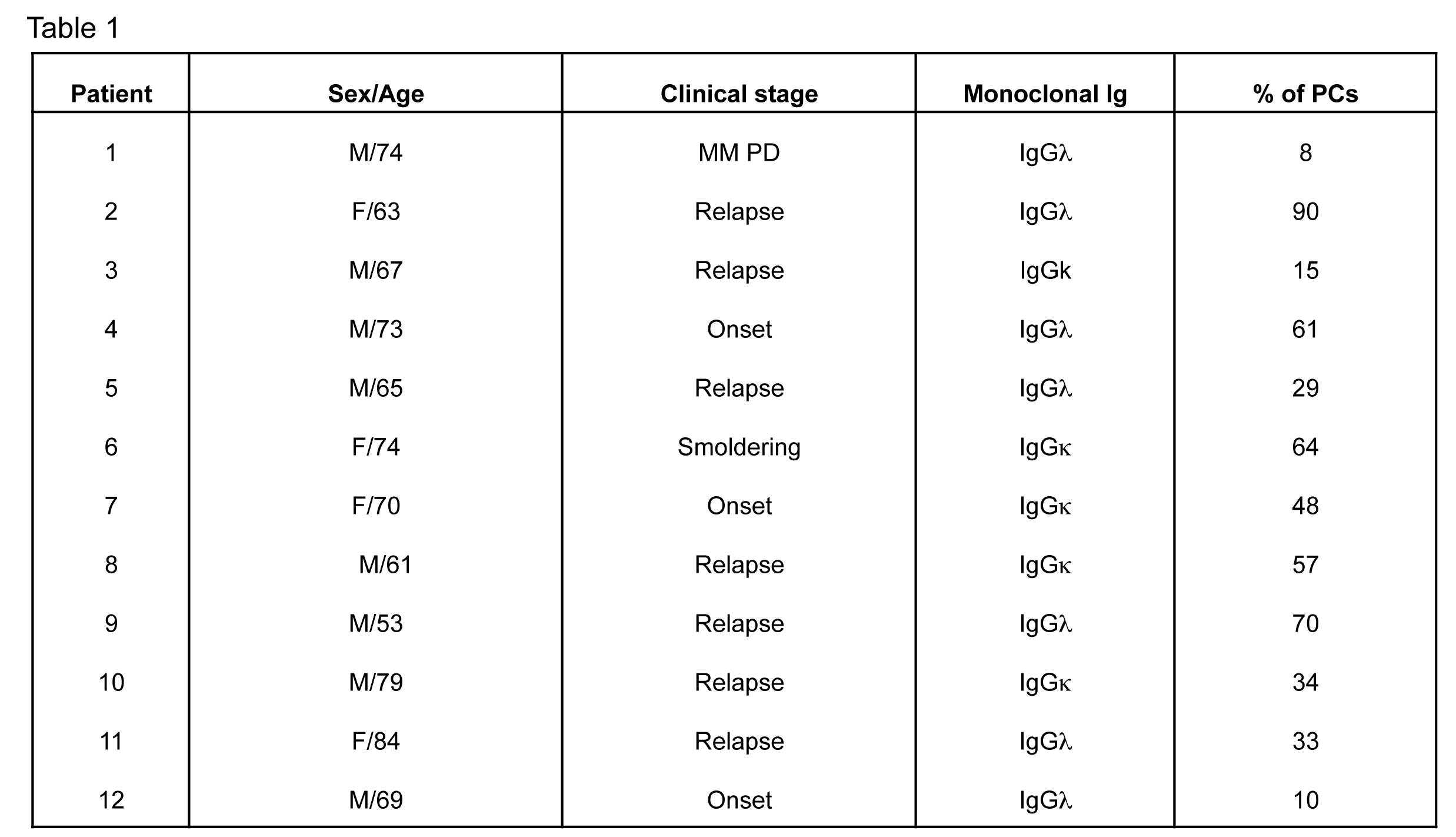

Supplement: Supplementary Table 1 — Clinical parameters of MM patients used for the analysis of GAS6 in condition medium derived from BMSCs and BM plasma. Patients were classified according to Durie and Salmon’s Staging System. [file Image_3.tif]

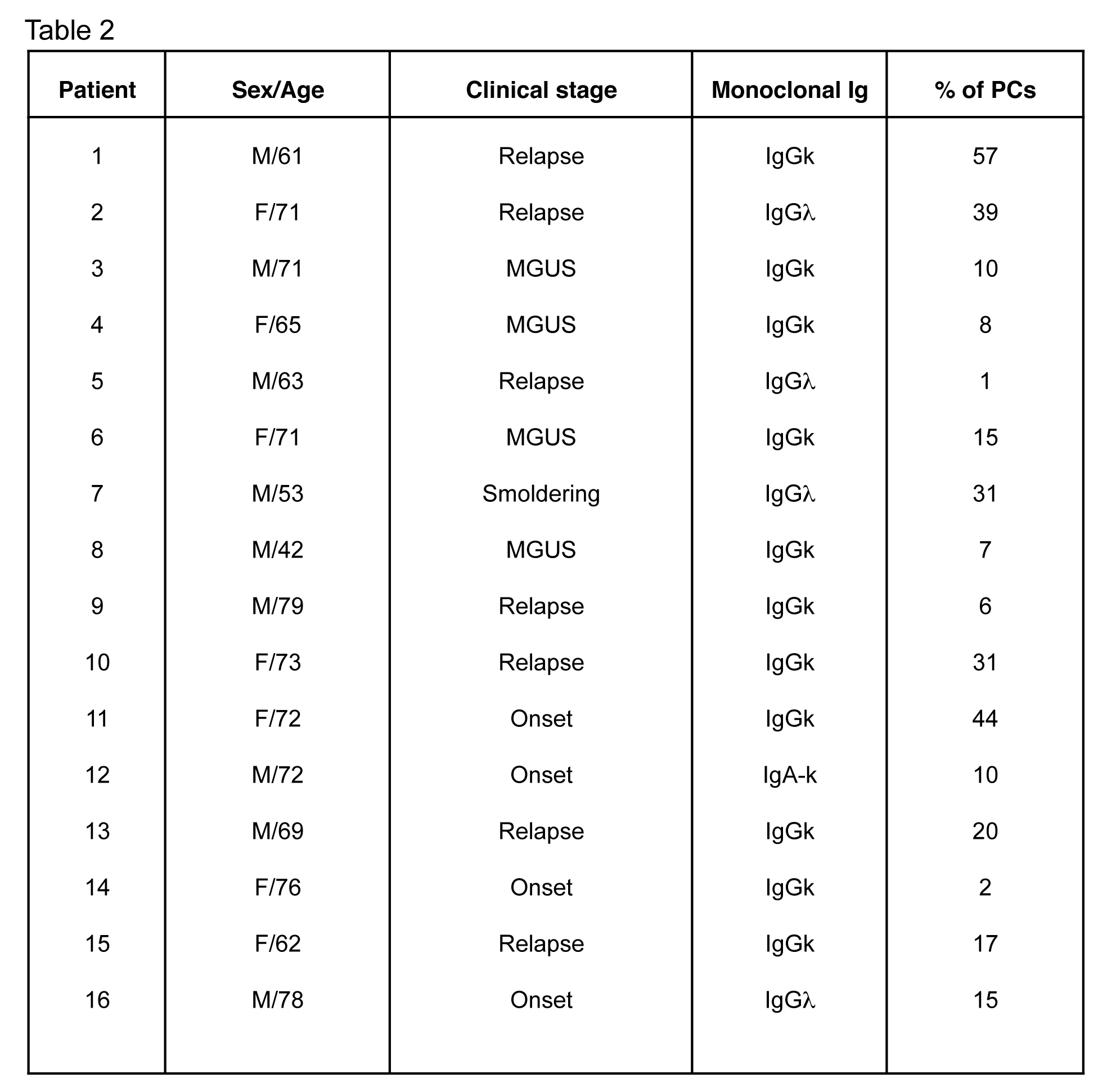

Supplement: Supplementary Table 2 — Clinical parameters of MM patients used for the analysis of MICA surface expression and mRNA. Patients were classified according to Durie and Salmon’s Staging System. [file Image_4.tif]
